# Corepressors SsnF and RcoA Regulate Development and Aflatoxin B_1_ Biosynthesis in *Aspergillus flavus* NRRL 3357

**DOI:** 10.3390/toxins14030174

**Published:** 2022-02-25

**Authors:** Xiaoyun Ma, Yiran Jiang, Longxue Ma, Shujuan Luo, Haolan Du, Xu Li, Fuguo Xing

**Affiliations:** Key Laboratory of Agro-Products Quality and Safety Control in Storage and Transport Process, Ministry of Agriculture and Rural Affairs, Institute of Food Science and Technology, Chinese Academy of Agricultural Sciences, Beijing 100193, China; xiaoyunma29@foxmail.com (X.M.); jiangyiranjyr@163.com (Y.J.); longxuem@foxmail.com (L.M.); luoshujuanjuan@163.com (S.L.); duhaolan-josh@foxmail.com (H.D.)

**Keywords:** *Aspergillus flavus*, Aflatoxin B_1_, carbon catabolite repression, SsnF, RcoA

## Abstract

*Aspergillus flavus* is a saprophytic fungus that can be found across the entire world. It can produce aflatoxin B_1_ (AFB_1_), which threatens human health. CreA, as the central factor in carbon catabolite repression (CCR), regulates carbon catabolism and AFB_1_ biosynthesis in *A. flavus*. Additionally, SsnF-RcoA are recognized as the corepressors of CreA in CCR. In this study, *ssnF* and *rcoA* not only regulated the expressions of CCR factors and hydrolase genes, but also positively affected mycelia growth, conidia production, sclerotia formation, and osmotic stress response in *A. flavus*. More importantly, SsnF and RcoA were identified as positive regulators for AFB_1_ biosynthesis, as they modulate the AF cluster genes and the relevant regulators at a transcriptional level. Additionally, the interactions of SsnF-CreA and RcoA-CreA were strong and moderate, respectively. However, the interaction of SsnF and RcoA was weak. The interaction models of CreA-SsnF, CreA-RcoA, and SsnF-RcoA were also simulated with a docking analysis. All things considered, SsnF and RcoA are not just the critical regulators of the CCR pathway, but the global regulators involving in morphological development and AFB_1_ biosynthesis in *A. flavus*.

## 1. Introduction

*Aspergillus favus* is an aerobic saprophytic fungus found in tropical and subtropical regions. It is an opportunistic phytopathogen, found in agro-products in pre- and post-harvest environments, such as those of maize, peanuts, and cottonseeds [1,2]. More importantly, *A. flavus* biosynthesizes several well-known polyketide mycotoxins, including aflatoxin B_1_ (AFB_1_) and aflatoxin B_2_ (AFB_2_), which threatens human and animal health [2]. AFB_1_ has the strongest carcinogenicity, teratogenicity, and toxicity [3], playing a causative role in global hepatocellular carcinoma cases [4]. In addition, AFB_1_ contamination of food and feed also results in significant economic losses and trade obstacles [5].

AF’ 30 biosynthetic genes, located in the #54 cluster, are responsible for AF’s biosynthesis, and acetyl-CoA and malonyl-CoA, as the AF’s precursors were catalyzed via polyketide synthase (AflC) [6]. The cluster-specific regulators, AflR and AflS, directly bind to the promoter regions and regulate the transcriptional expressions of AF’s biosynthetic genes [7]. Different environmental conditions, such as carbon sources, nitrogen sources, pH, light, temperature, water activity, phenolic compounds, lipids, and oxidative stress can also affect AF’s production [8]. Moreover, several global regulators, responding to diverse environmental changes, can, directly or indirectly, regulate the fungal metabolism of AF biosynthesis [6]. The source of carbon may be the most critical environmental factor for *Aspergillus*, as it supplies the energy for fungal development and is the basic carbon unit for secondary metabolites production [9].

Carbon catabolite repression (CCR) is a critical regulatory pathway for preferential carbon utilization. It is involved in fungal growth development and secondary metabolisms [9]. In *Saccharomyces cerevisiae*, the C_2_H_2_ family transcriptional regulator, Mig1, is modified by diverse CCR factors, and then regulates the targets by binding to the promoter regions of downstream target genes [10]. In filamentous fungi, CreA is homologous with Mig1 and serves its function after diverse post-transcriptional regulation [11]. For instance, the CreD-HulA ubiquitination ligase complex helps CreA ubiquitination, while the CreB-CreC deubiquitination (DUB) complex removes ubiquitin from CreA [12,13]. Several phosphatases and kinases, including Snf1, SchA, Reg1, and Glc7, control the CreA phosphorylation level, and furthermore, affect its localization and functionality [11]. A thorough study of CCR is important to understand the functions and regulations of CreA and is helpful in revealing the manner in which fungi utilize carbon sources.

Cyc8 (homologous with Ssn6 or SsnF) and Tup1 (homologous with RcoA) are critical transcriptional repressors in CCR. They are involved in nutrient uptake, mating type, and other metabolisms in *S. cerevisiae* [14]. In yeast, the recruit proteins Cyc8 and Tup1 are recruited by Mig1, and regulate the target’s expressions by binding to the promoter regions of downstream genes, such as diverse hydrolase genes [15]. Another study suggested that Mig1 could combine with the corepressors Tup1 and Ssn6 to form a trimer complex, then, together, bind to the target genes’ promoter regions [10]. In *A. nidulans*, the regulation of the SsnF-RcoA complex seems more complicated. Deletion of RcoA does not eliminate CCR, but it does alter the chromatin structure of carbon catabolite repressible promoters, and deletion of SsnF is lethal [16]. Therefore, the regulations, functions, and relationships among CreA, RcoA, and SsnF are still obscure. There are very few studies about RcoA and SsnF in *Aspergillus*.

CreA, as the central regulator of CCR, is also regarded as a positive regulator for AF biosynthesis [17]. SsnF and RcoA are recruited by CreA/Mig1, but few studies have covered the physical interactions among CreA, SsnF, and RcoA. Additionally, no report has revealed how SsnF and RcoA affect AFB_1_ production in *A. flavus*. In this study: a null-deletion mutant of *ssnF* and *rcoA* was generated, the effects of SsnF and RcoA upon the growth, development, and AFB_1_ biosynthesis in *A. flavus* were partly revealed, and the interaction moderns between SsnF/RcoA and CreA were verified and reconstructed. Our results provide a comprehensive analysis of SsnF and RcoA in *A. flavus* and contribute to a better understanding of the relationship between the CCR pathway and AFB_1_ biosynthesis.

## 2. Results

### 2.1. Bioinformatic Analyses and Deletion Mutant Constructions of SsnF and RcoA in A. flavus

**Figure 1:**
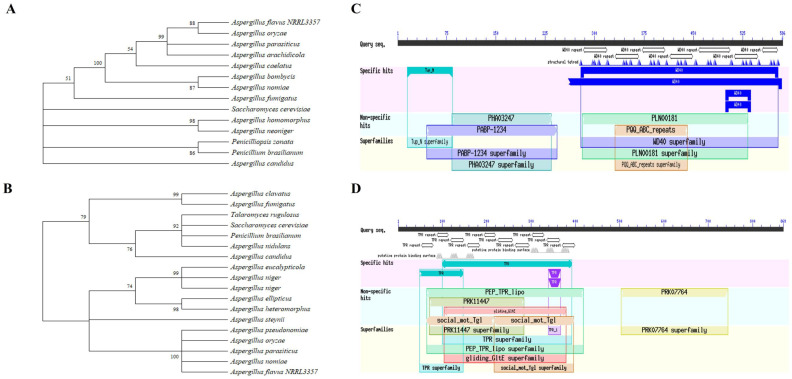
Bioinformatics analyses of the recruiter proteins SsnF and RcoA: (**A**) The phylogenetic trees of SsnF; (**B**) the phylogenetic trees of RcoA; (**C**) the protein structural analyses of SsnF (**D**) the protein structural analyses of RcoA.

The recruiter proteins SsnF (AFLA_134730, 869 amino acid) in *A. flavus* is homologous with Cyc8/Ssn6 in *S. cerevisiae* or *A. nidulans*, and the transcriptional repressor RcoA (AFLA_054810, 586 amino acid) is derived from TupA in *S. cerevisiae* [16,18]. The phylogenetic trees were constructed with the amino acid sequences of SsnF and RcoA. As Figure 1 shows, SsnF in *A. flavus* NRRL 3357 was most similar to *A. oryzae*, while RcoA was shown to be highly homologous with *A. pseudonomiae*, *A. oryzae*, *A. parasiticus*, and *A. nomiae* (Figure 1A,B). Domain analyses revealed that SsnF was consistent with the tetracripeptide repeat (TPR) domain and the PRK07764 superfamily domain in N- and C- terminals, respectively. The Tup_N and WD40 superfamily domains were recognized in the N- and C- terminals of RcoA protein, respectively (Figure 1C,D). For a better understanding of the regulations and functions of SsnF and RcoA, the *ssnF* and *rcoA* mutants, and their complementary strains, were generated with homologous recombination. The derived strains were verified by PCR amplification (Figure S2).

### 2.2. SsnF and RcoA Are Necessary for Vegetative Growth and Fungal Development in A. flavus

**Figure 2:**
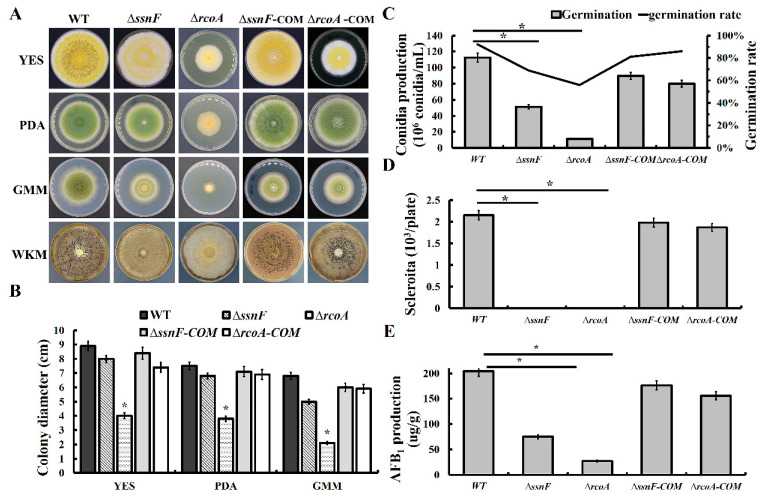
The diverse phenotypic variations of WT, *ssnF,* and *rcoA* mutants, and the complementary strains: (**A**) the colony morphology of different strains on YES, PDA, GMM, and WKM media; (**B**) the growth rate of different strains; (**C**) the conidia production of different strains; (**D**) sclerotia formation of different strains; (**E**) AFB_1_ biosynthesis of different strains. Bars represent SD from three independent experiments with three replicates. * shows a significant difference at *p* < 0.05.

Compared with the wild-type strain (WT), Δ*rcoA* growth rates were significantly decreased in yeast extract sucrose (YES), potato dextrose agar (PDA), and glucose minimum medium (GMM), but the colony diameters of Δ*ssnF* were only slightly less than WT, without statistical significance (Figure 2A,B). The colony color of Δ*ssnF* and Δ*rcoA* were irregularly faded (Figure 2A), suggesting that the conidia pigment formations were disturbed. Additionally, conidia production of Δ*ssnF* and Δ*rcoA* was significantly lower than WT, but the germination rates of different strains were not significantly changed. (Figure 2C). The sclerotia formation was completely blocked in Δ*ssnF* and Δ*rcoA*, implying SsnF and RcoA might be essential for sclerotia production in *A. flavus* (Figure 2A,D). Additionally, the complementary strains recovered the WT phenotypes (Figure 2).

### 2.3. SsnF and RcoA Regulate AFB_1_ Biosynthesis by Modulating the Transcriptional Expressions of AF Cluster Genes and AF Related Regulators

**Figure 3:**
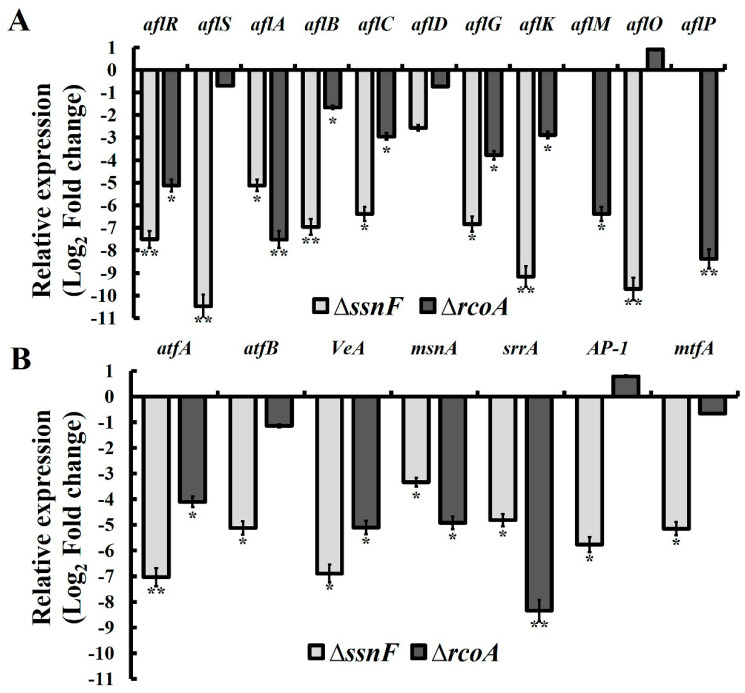
RT-qPCR analyses of AFB_1_ related gene expressions in Δ*ssnF*, Δ*rcoA,* and WT: (**A**) expressions of AF biosynthesis cluster genes; (**B**) the expressions of diverse global regulators. The expressions of the examined genes were normalized to the expression of the *actin* gene * and ** show a significant difference at *p* < 0.05 and *p* < 0.01, respectively.

AFB_1_ production in Δ*ssnF* and Δ*rcoA* were only about 1/3 and 1/7 of WT, respectively (Figure 2E), suggesting that both SsnF and RcoA could positively regulate AFB_1_ biosynthesis in *A. flavus*. Additionally, AFB_2_ production in WT was much less than that of AFB_1_, and AFB_2_ production in WT was also significantly reduced in Δ*ssnF* and Δ*rcoA*. The decrease of AFB_2_ in Δ*ssnF* was more drastic (Figure S3). Upon further investigation, the expressions of AF’s biosynthetic genes were analyzed by RT-qPCR. Both *aflR* and *aflS*, the pathway-specific regulators, were significantly down-regulated in Δ*ssnF* and Δ*rcoA*, the exception being that *aflS* in Δ*rcoA* showed no significant variation (Figure 3A). Additionally, several critical AF biosynthetic structural genes, such as *aflA*, *aflB*, *aflC*, *aflG*, *aflK*, *aflM*, *aflO,* and *aflP*, were significantly down-regulated in Δ*ssnF* and Δ*rcoA*, while the expressions of *aflD* did not, in a statistically significant manner, decrease in two mutants (Figure 3A). Expressions of the AF related TFs were also detected in this study. All mentioned TFs were significantly down-regulated in two mutants, but *atfB*, *AP-1,* and *mtfA* were not significantly changed in Δ*rcoA* (Figure 3B). Taken together, SsnF and RcoA could regulate the transcriptional expressions of AFB_1_ biosynthetic genes and AFB_1_ related TFs, before then affecting AFB_1_ production.

### 2.4. SsnF and RcoA Affect the Expressions of Hydrolase Genes by Regulating CCR Pathway Factors

**Figure 4:**
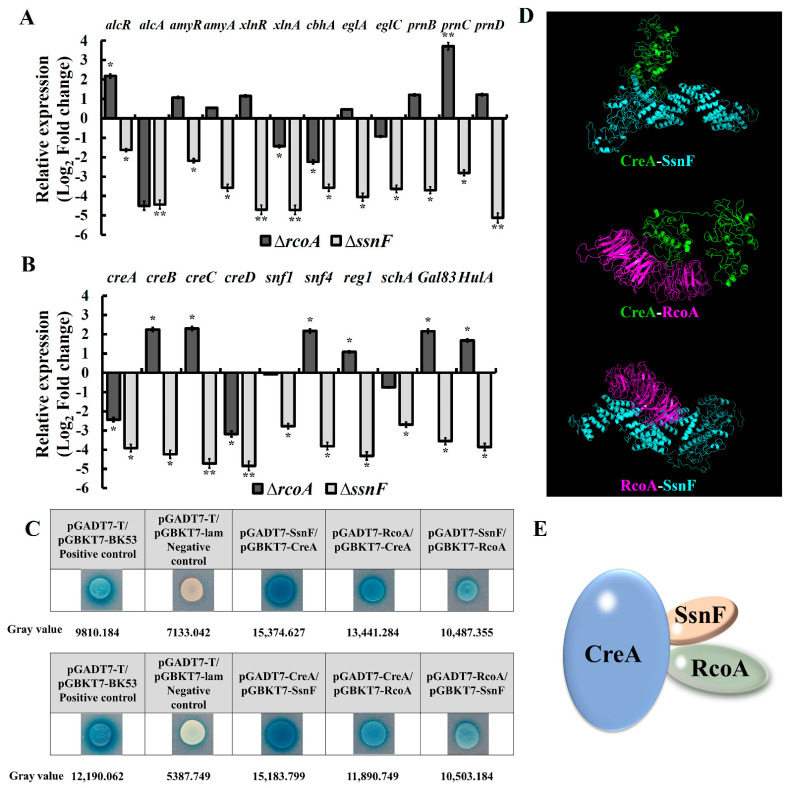
The effects of SsnF and RcoA on CCR factors in *A. flavus*: (**A**) RT-qPCR analyses of the hydrolase related genes; (**B**) RT-qPCR analyses of CCR factors (The expressions of the examined genes were normalized to the expression of *actin* gene * and ** show a significant difference at *p* < 0.05 and *p* < 0.01, respectively); (**C**) the interaction levels of CreA, SsnF, and RcoA, with the numbers representing the gray values of the colonies’ color; (**D**) the physical interactions of CreA, SsnF, and RcoA, verified by autodocking; (**E**) the predicted interaction model of CreA, SsnF, and RcoA.

The transcriptional expressions of the hydrolase genes are directly regulated by CCR pathway factors [19]. In this study, the expressions of hydrolase genes and the specific regulators, such as *alcR*, *alcA*, *amyR*, *amyA*, *xlnR*, *xlnA*, *cbhA*, *eglA*, *eglC*, *prnB*, *prnC,* and *prnD* were significantly decreased in Δ*ssnF* (Figure 4A). However, in Δ*rcoA*, only *xlnA* and *cbhA* were significantly down-regulated, while *alcR* and *prnC* were shown to be significantly up-regulated (Figure 4A). All CCR pathway genes were significantly down-regulated in Δ*ssnF* (Figure 4B). However, as Figure 4B shows, while *creA* and *creD* were significantly decreased, the majority of CCR genes (*creB*, *creC*, *snf4*, *reg1*, *gal83,* and *hula*) were significantly increased in Δ*rcoA* (Figure 4B). All of the above results imply that SsnF might be the more critical and crucial regulator, rather than RcoA in CCR regulations.

Furthermore, the interactions between CreA, SsnF, and RcoA were investigated by yeast two-hybrid (Y2H). As Figure 4 shown, CreA strongly interacted with SsnF, but moderately interacted with RcoA, while the interaction between SsnF and RcoA was relatively slight (Figure 4C). As shown in Figure 4D, the physical interactions of CreA, SsnF, and RcoA were verified by autodocking, but we still failed to construct the tri-proteins interaction model (Figure 4D). Based on the above information, the predicted complex of CreA, SsnF, and RcoA was generated as Figure 4E. Taken together, SsnF and RcoA could not only be interacted with and recruited by CreA, but also directly modulate CreA expression. SsnF and RcoA are critical to maintaining the regular function of the CCR pathway.

### 2.5. SsnF and RcoA Are Associated with the Response to Osmotic Stress in A. flavus

**Figure 5:**
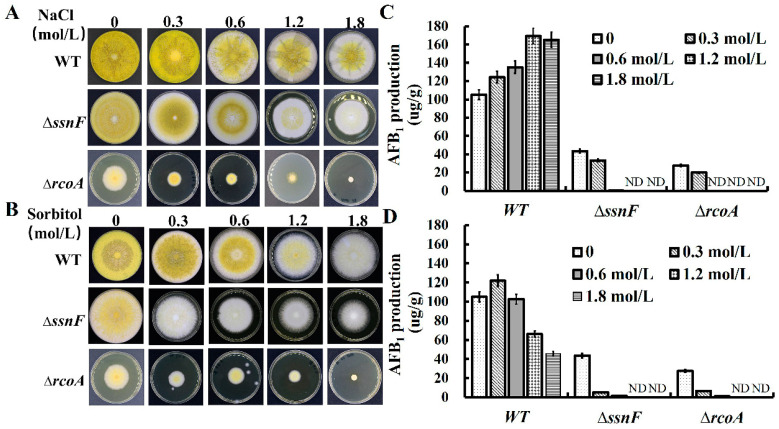
The effect of *ssnF* and *rcoA* on osmotic stress of *A. flavus*: (**A**) the WT, Δ*ssnF,* and Δ*rcoA* were cultivated on YES media with different NaCl concentrations; (**B**) the WT, Δ*ssnF,* and Δ*rcoA* were cultivated on YES media with different D-sorbitol concentrations; (**C**) AFB_1_ productions of WT and mutant strains at different NaCl concentrations; (**D**) AFB_1_ productions of WT and mutant strains at different D-sorbitol concentrations. Bars represent SD from three independent experiments with three replicates. ND stands for the signals below the detection limit.

In the previous study, the Ssn6-Tup1 complex participated in the regulation of osmotic stress responses in genes in *S. cerevisiae*, with several CCR factors involved in osmotic stress regulations [20]. As such, we also evaluated the effect of *ssnF* and *rcoA* on osmotic stress in this study. The WT, Δ*ssnF,* and Δ*rcoA* were cultivated on YES media with different concentrations of NaCl and D-sorbitol (Figure 5A,B). With an increase in NaCl, mycelia growth and conidia productions of WT and Δ*ssnF* were slightly decreased (Figure 5A and Figure S5A). However, the fungal development of Δ*rcoA* was severely impaired, especially in 1.8 mol NaCl/L conditions, and the growth of Δ*rcoA* was completely inhibited (Figure 5A and Figure S5A). AFB_1_ production in WT improved with the increase of NaCl, but AFB_1_ biosynthesis of Δ*ssnF* and Δ*rcoA* were significantly suppressed by NaCl. Δ*ssnF* and Δ*rcoA* hardly biosynthesized AFB_1_ at all in conditions containing more than 0.6 mol NaCl/L concentrations (Figure 5C). The effect of D-sorbitol upon the growth of WT and mutants was similar to that of NaCl (Figure 5B and Figure S5B). AFB_1_ production in WT decreased with more than 0.3 mol D-sorbitol/L D-sorbitol, but AFB_1_ production of Δ*ssnF* and Δ*rcoA* was almost completely inhibited with the addition of D-sorbitol (Figure 5D). All these findings suggested that Δ*ssnF* and Δ*rcoA* were more sensitive to NaCl and D-sorbitol and that SsnF and RcoA could be involved in osmotic stress response.

## 3. Discussion

CCR, as the most crucial regulatory system, has been extensively studied in diverse fungi. In the central TF, CreA/Mig1 represses the expression of hydrolase genes by binding their promoter regions in limited glucose conditions [12]. RcoA and SsnF, homologous with Tup1 and Ssn6 in *S. cerevisiae*, respectively, are recruited by CreA in *Candida albicans* and *A. nidulans* [16,18]. In *S. cerevisiae*, Mig1 binds to the glucose-repressive genes’ promoters and inhibits their transcriptions by recruiting the corepressor complex Ssn6-Tup1 [21]. As such, we initially speculated that the hydrolase genes’ transcriptions would be up-regulated with the deletion of *ssnF* or *rcoA*. Contrarily, all of the detected genes significantly decreased. Δ*ssnF*, *aclA*, *xlnA,* and *cbhA* were also significantly down-regulated in Δ*rcoA* (Figure 4A). Furthermore, expressions of *creA* significantly decreased in two mutants (Figure 4A). It seemed paradoxical that both the transcriptional repressor CreA and the downstream hydrolase genes were down-regulated at the same time. In *Trichoderma reesei*, diverse hydrolase genes, such as *cbh1*, *egl1*, *bgl1, and xyr1* were drastically reduced in *cyc8* or *tup1* deleted strains, through which the Cyc8-Tup1 complex was identified as the coactivator for xylanase production in *T. reesei*, rather than the corepressor [22]. In fact, RcoA and SsnF frequently modulate downstream gene expressions independently of CCR regulation. Cyc8 in yeast regulates *GAL1* without Mig1 recruitment, and deletion of *cyc8* does not elevate *GAL1* expression [15,23]. In addition, Cyc8-Tup1 could directly interact with histones H3 and H4, influencing their acetylation [24]. Wang et al. suggested that the Cyc8-Tup1 complex interacts with the SAGA histone acetylase, and thus, participates in the remodeling of nucleosomes to activate the cellulase genes’ transcriptions [22]. In *A. nidulans*, RcoA is also essential for nucleosome positioning in the promoter region [16]. Taken together, SsnF and RcoA might not only be corepressors in CCR, but also positive regulators, independent of CreA regulation.

The expressions of CCR factors were especially varied in Δ*ssnF* and Δ*rcoA*. All CCR genes in Δ*ssnF* were drastically decreased (Figure 4B). Therefore, we suggested SsnF and RcoA could be global regulators affecting CCR factor expressions, more than members of the CCR pathway. Additionally, RcoA and SsnF could be involved with several distinct features of fungi [22]. In yeast, more than 150 genes were repressed by the Ssn6-Tup1 complex, and diverse phenotypical variations were exhibited in *ssn6* and *tup1* mutants [25]. Gong et al. found that Tup1 participated in the filamentation of *C. tropicalis* [26], and deletion of *rcoA* resulted in gross defects in vegetative growth, asexual spore production, and sterigmatocystin biosynthesis in *A. nidulans* [27]. Similarly, the mycelia growth, conidia production, and AFB_1_ biosynthesis of *A. flavus* were severely impaired in Δ*rcoA*, and the sexual sclerotia formation was completely eliminated in Δ*ssnF* and Δ*rcoA* (Figure 1). Deletion of *rcoA* showed more severely impaired morphology and development than Δ*ssnF* in *C. tropicalis*, *T. reesei*, *A. nidulans,* and *A. flavus*, suggesting that RcoA might be indispensable for normal fungal development [22,26,27]. Similarly in this study, the inhibition of mycelia growth in Δ*rcoA* was more drastic than in Δ*ssnF*. We also noticed that several developments relating to TFs, AtfA, AtfB, VeA, AP-1, MsnA, MtfA, and SrrA were down-regulated in Δ*ssnF* and Δ*rcoA* (Figure 3B). These global TFs respond to different environmental stresses, coordinate sexual and asexual development, and modulate downstream gene transcriptions through direct binding [6]. VeA is especially required, as the central regulator of the heterotrimeric complex, for sclerotia formation and conidia development [8]. It is reasonable to speculate that SsnF and RcoA might regulate *A. flavus* development by affecting the expression of *veA*. Furthermore, Δ*ssnF* and Δ*rcoA* were shown to be more sensitive to osmotic stress, and Δ*rcoA* was almost completely suppressed by 1.2 M NaCl (Figure 5). The vulnerable phenotypes of mutants might be contributing to the drastic decrease in the expressions of AtfB and AP-1, to which these two regulators are essential to osmotic stress and membrane stimulus [28,29]. Taken together, SsnF and RcoA could regulate several global TFs, then affect downstream gene expressions, and, subsequently, modulate fungal growth and development. SsnF and RcoA would also be the pleiotropic global regulators involved in diverse biological processes.

As the most critical features of *A. flavus*, AFB_1_ biosynthesis was also affected by SsnF and RcoA. Both *ssnF* and *rcoA* mutants exhibited defective AF biosynthesis in the YES medium, and Δ*rcoA* was less than 1/7 AFB_1_ production of WT (Figure 2E). Additionally, several AF biosynthetic genes were significantly down-regulated in Δ*ssnF* and Δ*rcoA* (Figure 3A), implying that SsnF and RcoA could positively affect AF production by regulating AF structural genes expression. AflR and AflS could bind to the promotors of AF’ biosynthetic genes, positively regulating AF gene transcriptional expressions [7]. Our results showed that *aflR* and *aflS* were also significantly decreased in Δ*ssnF* and Δ*rcoA* (Figure 3A). Similarly, the *rcoA* deleted strain showed the absent expression of *aflR* in *A. nidulans* [27]. Several oxidative stresses, such as TFs, AtfA, AtfB, AP-1, and SrrA were down-regulated in Δ*ssnF* and Δ*rcoA* (Figure 3B), with which these global TFs could positively regulate AF gene expressions [28]. Consequently, SsnF and RcoA could regulate AF cluster gene expressions, depending on these global TFs. Besides this, CreA was also reported as a positive regulator for AF cluster genes. A CreA binding motif (5′-SYGGRG-3′) was discovered in the promotor regions of several AF cluster genes, including *aflR* and *aflS* [17,30]. SsnF and RcoA could also affect AF biosynthesis via CreA. Therefore, SsnF and RcoA might be necessary for AF biosynthesis and the regulation of AF cluster gene expressions at a transcriptional level. Additionally, several hydrolase genes were down-regulated in Δ*ssnF* and Δ*rcoA*, through which the utilization of carbon sources might be disturbed in two mutants. Carbon catabolism provides the energy and raw materials for AFB_1_ biosynthesis, so decreasing carbon catabolism might also lead to AFB_1_ down-regulation.

The interactions among CreA, SsnF, and RcoA were also investigated in our study. In *S. cerevisiae*, SsnF and RcoA, as the integrated protein complex, are physically associated with a 1:4 ratio [31]. However, a relatively weak interaction between SsnF and RcoA was recorded in this study (Figure 4D). Tartas et al. found the most blatant interaction to be that between the N-terminal non-TPR region of Tup1 and the glutamine-rich tail of Ssn6 in *S. cerevisiae* [32], implying that the interaction of SsnF and RcoA would occur between the two protein domains. Furthermore, the interaction between SsnF and CreA was stronger than that of RcoA and CreA (Figure 4D), and the expressed variations of downstream genes were more drastic in Δ*ssnF* than Δ*rcoA* (Figure 3 and Figure 4). Therefore, we believed that SsnF would play a more critical role in the SsnF-RcoA complex. Similarly, Hicks et al. also believed that RcoA played a weak role in CCR regulon and its relevant metabolism [27]. However, deletion of *tup1*/*rcoA* demonstrated more severe impairments in different fungi, suggesting that RcoA might be more essential for fungal development [22,26,27]. As such, the functions of SsnF and RcoA do not overlap. SsnF serves a critical function in downstream gene regulation, while RcoA is theorized to be more indispensable to normal fungal growth and development.

**Figure 6:**
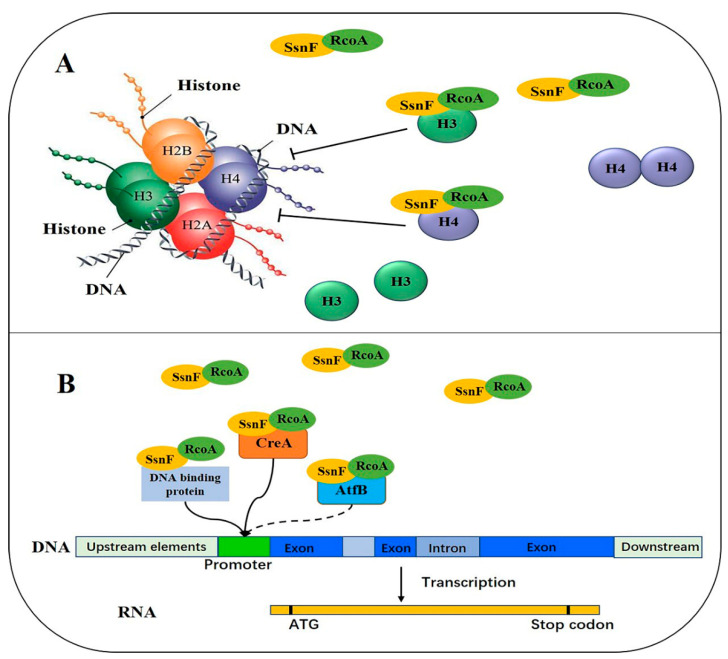
A schematic model of SsnF-RcoA complex: (**A**) SsnF-RcoA directly interacting with histones H3 and H4, influencing their acetylation; (**B**) SsnF-RcoA interacting with several DNA-binding proteins and then regulating the downstream gene transcription.

Taking all of this information into account, we theorize that SsnF-RcoA might not only be the global corepressor, but also the coactivator in *A. flavus*, physically interacting with several DNA-binding proteins, regulating downstream gene expressions, coordinating growth, development, and AFB_1_ biosynthesis (Figure 6).

## 4. Conclusions

In this study, SsnF and RcoA positively regulate mycelia growth, conidia production, and sclerotia formation in *A. flavus*. Additionally, SsnF and RcoA might be the positive regulators for AFB_1_ biosynthesis through the modulation of AF related genes at the transcriptional level. The positive effects of SsnF and RcoA on transcriptional expressions of CCR factors and hydrolase genes were also observed. Additionally, a protein interaction model of SsnF, RcoA, and CreA was constructed based on the Y2H and protein docking analyses. Taken together, the SsnF-RcoA complex could not only play a role in the CCR pathway as the corepressor but could also positively regulate downstream genes by interacting with other global regulators. Our research partly reveals the regulatory mechanism of the SsnF-RcoA complex with regards to AFB_1_ biosynthesis and is helpful to illustrate the relationship between the CCR pathway and AF biosynthesis.

## 5. Materials and Methods

### 5.1. Strains and Culture Conditions

The strain *A. flavus* NRRL 3357 was kindly provided by Prof. Wenbing Yin of the Institute of Microbiology, Chinese Academy of Sciences. Potato Dextrose Agar (PDA), Glucose Minimum Medium (GMM), and Yeast Extract Sucrose (YES) were prepared, as previously stated, and were used to evaluate growth rates, conidial development, and AFB_1_ production, respectively [17]. Fungal development was observed after 7 days of cultivation at 28 °C in the dark. The sclerotia formation was evaluated at 37 °C in the dark for 14 days via the Wickerham (WKM) medium [33].

### 5.2. Sequence Analyses and Phylogenetic Tree Construction

The *ssnF* (AFLA_134730) and *rcoA* (AFLA_054810) nucleotide sequences and protein sequences (EED50710.1 and ID EED51218.1, respectively) were obtained from the National Center for Biotechnology Information Database (NCBI). The amino acid sequences of SsnF and RcoA orthologs proteins were also downloaded from the NCBI, were aligned by ClustalW, and the phylogenetic tree was constructed by MEGA 5.1 software [34].

### 5.3. Construction of the Deletion Cassette and Complementary Strains

The deletion cassette was constructed using the fusion PCR [17]. Primers used in this study are listed in Table S1. The fusion PCR products with the *pyrG* marker were then transformed into the protoplasts of *A. flavus* through a PEG-mediated transformation [35]. The transformants were selected via the uracil-lacking medium and were verified via diagnostic PCR (Figures S1 and S2).

The complementary strains were generated with similar methods. Using cDNA as the template, the CDS fragments of *ssnF* and *rcoA* were amplified. According to Figure S2, the CDS fragments and the marker fragment were fused with upstream and downstream sequences of target genes. The complementary vectors were transformed into protoplasts of Δ*ssnF* and Δ*rcoA* to generate complementary strains (Δ*ssnF*-COM and Δ*rcoA*-COM).

### 5.4. The Extraction and Detection of AFB_1_ Production

The methods of AFB_1_ extraction and detection were followed by Liang et al. [36] with some modifications. The conidia suspension of *A. flavus* (10^7^ conidia/mL) was inoculated on YES plates at 28 °C in dark for 7 days. Three agar disks (about 0.5 g) were obtained by a puncher, transferred to a 10 mL centrifuge tube, and then extracted with 3 mL methanol for 1 h. After 30 min of ultrasonic vibration at 40 khz, 1 mL of the supernatant solution was filtered through a 0.22 µm filter. For AFB_1_ detection, HPLC analysis was performed on an Agilent HPLC system (Agilent Technologies, Santa Clara, CA, USA) with an Agilent 1260 fluorescence detector (λexc 360 nm; λem 440 nm), a postcolumn derivation system, and an Agilent TC-C18 column (250 mm × 4.6 mm, 5 μm particle size, Agilent). The mobile phase (water: methanol, 3:7) was pumped at a flow rate of 1 mL/min, and the retention time of AFB_1_ was about 5.7 min. The mean recovery was calculated by spiking YES broth at different levels ranging from 1 to 100 ng/g of AFB_1_ and was estimated at 95.2 ± 8.4%. The limit of the lowest detection was 1 ng/g, and the limit of quantitation was 10 ng/g. The linearity range of the used method was 0.1–100 μg/mL (r ≥ 0.9990).

### 5.5. RNA Extraction and RT-qPCR Analysis

The mycelia were harvested by shaking cultivation in YES broth, 28 °C, 180 rpm for 3 days. Total RNA was extracted from wild-type and mutant strains according to the instructions of RNApure Total RNA Kit (Aidlab Biotechnologies Co., Ltd., Beijing, China), and the RNA quality was checked by agarose gel and Agilent 2100 Bioanalyzer (Agilent, Santa Clara, CA, USA). The removal of gDNA and synthesized first-strand cDNA was performed using the cDNA synthesis Kit (TIANGEN, Beijing, China).

The cDNA template was diluted to 100 ng/μL by a trace nucleic acid analyzer, and the reaction system was prepared according to the instructions for use of the Power SYBR Green Master Mix Kit (TIANGEN, Beijing, China). Using the QuantStudio 6 Flex (Applied Biosystems, Carlsbad, CA, USA) qPCR system, the *actin* gene was used as a reference to normalize the target gene, and gene expression was calculated via the 2^−ΔΔCt^ method. The primers used for qPCR analysis are listed in Table S2. Three independent biological replicates were produced for this study.

### 5.6. Yeast Two-Hybrid Assay

The CDS regions of *ssnF*, *rcoA,* and *creA* were amplified via total cDNA. Using the ClonExpress II One Step Cloning Kit (Vazyme Biotech, Nanjing, China), the CDS fragments were inserted into pGBKT7 and pGADT7, respectively. The constructed plasmids were sequenced in Sangon Biotech (Figure S4), then, pairwise, co-transformed into the Y2HGold cell by the Yeastmaker™ Yeast Transformation System 2 (630439, Takara, Dalian, China). All of the selective media, including SD (lacking leucine)/-Leu, SD (lacking tryptophan)/-Trp, SD (lacking leucine and tryptophan)/-Leu/-Trp, SD (lacking histidine, leucine, tryptophan, and adenine)/-His/-leu/-Trp/-Ade and X-α-gal were purchased from Coolaber (Beijing, China). The Y2H Gold cells containing pGBKT7-p53 and pGADT7-T were set as the positive control.

### 5.7. Analysis of Osmotic Stress on Different Mutants

The 10^6^ conidia of *A. flavus* was inoculated by the YES media with different stress agents at 28 °C for 7 days. NaCl and D-sorbitol were regarded as the stress agents in different final concentrations (0.3 M, 0.6 M, 1.2 M, and 1.6 M). The fungal development and AFB_1_ productions were measured as above.

### 5.8. Protein Docking Analysis

The SsnF, RcoA, and CreA protein structures were downloaded from the PDB Database (http://www.rcsb.org/pdb (accessed on 25 January 2022)). The autodocking software was used for the protein docking analysis (AutoDock_vina_1_1_2_win32), and the parameters were set as the default value [37,38]. The model of protein interaction was generated with the PyMol (2.7 Version) software [38].

### 5.9. Statistical Analysis

All experiments were repeated with three independent biological replicates. The results are presented as means with standard deviations. The student’s *t* test was applied to compare the differences of phenotypes and expressions with SPSS 12.0, and differences were marked with * and ** as *p* < 0.05 and *p* < 0.01, respectively.

### 5.10. Experimental Design

**Figure 7:**
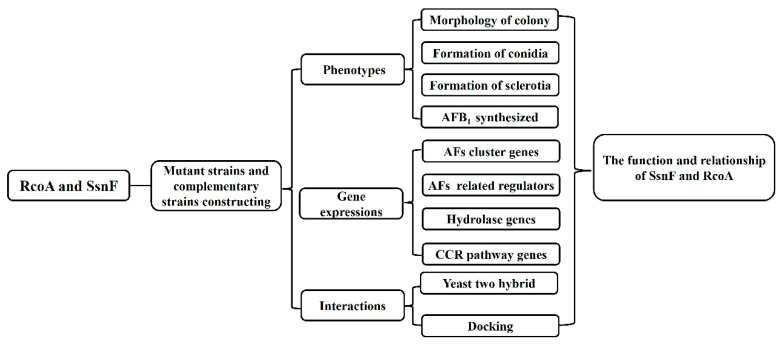
The flow chart of the experiment design in the study.

As shown in the Figure 7, the null-deletion mutants of RcoA and SsnF were generated first. Then, the phenotypes of mutants, including fungal growth, conidia development, sclerotia formation, and AFB_1_ production, were compared with WT. Furthermore, we attempted to illustrate the phenotype’s changes by detecting the relevant gene expressions. In addition, the protein interactions among SsnF, RcoA, and CreA were analyzed using Y2H and protein docking. Based on these experiments, we concluded that SsnF and RcoA should not only be the recruit proteins in the CCR pathway, but also participate in several regulations as global regulators.

## Data Availability

The data presented in this study are available in this article and Supplementary Materials.

## Supplementary Materials

The following supporting information can be downloaded at: https://www.mdpi.com/article/10.3390/toxins14030174/s1, Figure S1: The PCR products of *ssnF* and *rcoA* flanking fragments.; Figure S2: PCR verification of deletion and complementation strains; Figure S3: AFB_2_ biosynthesis of different strains. Figure S4: The acquirements of CDS fragments and the Y2H strains; Figure S5: The productions of conidia and AFB_2_ in different strains under osmotic stress; Table S1: PCR primers used in this study; Table S2: qPCR primers used in this study.
