# Supplementary material for: Corepressors SsnF and RcoA Regulate Development and Aflatoxin B1 Biosynthesis in Aspergillus flavus NRRL 3357
Source: Toxins (Basel). 2022 Feb 25;14(3):174. doi: 10.3390/toxins14030174 (PMC8954095; doi:10.3390/toxins14030174)
Supplement: Supplementary file 1 [file toxins-14-00174-s001.zip › toxins-1549110-SI.pdf]

Supplementary Materials: Corepressors SsnF and RcoA Regulate Development and Aflatoxin B_1 Biosynthesis in *Aspergillus flavus* NRRL 3357

Xiaoyun Ma, Yiran Jiang, Longxue Ma, Shujuan Luo, Haolan Du [1], Xu Li and Fuguo Xing

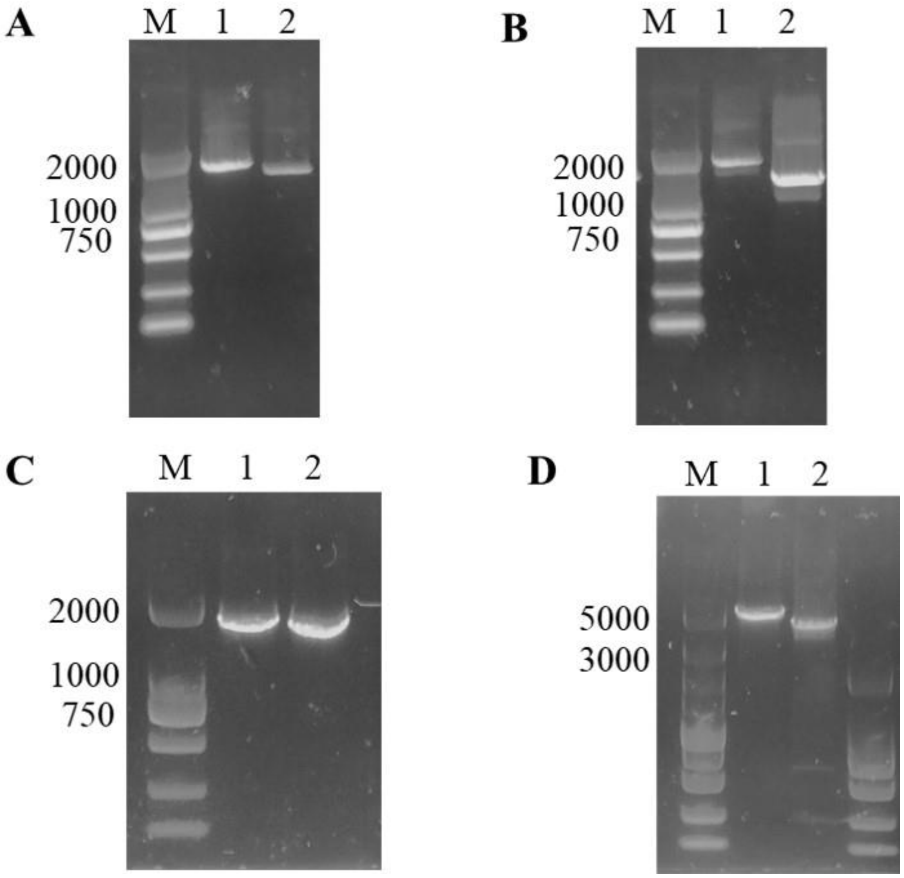

Figure S1. The PCR products of *ssnF* and *rcoA* flanking fragments. (A) Lane M: DL5000 Marker, Lane 1: 5′flanking region of *ssnF*, Lane 2: 3′ flanking region of *ssnF*; (B) Lane M: 2000 Marker; Lane 1: 5′flanking region of *rcoA*, Lane 2: 3′ flanking region of *rcoA*; (C) Lane M: 2000 Marker, Lane 1-2: *pyrG* gene; (D) Lane M: 5000 Marker, Lane 1: deletion cassette of *ssnF*, Lane 2: deletion cassette of *rcoA*.

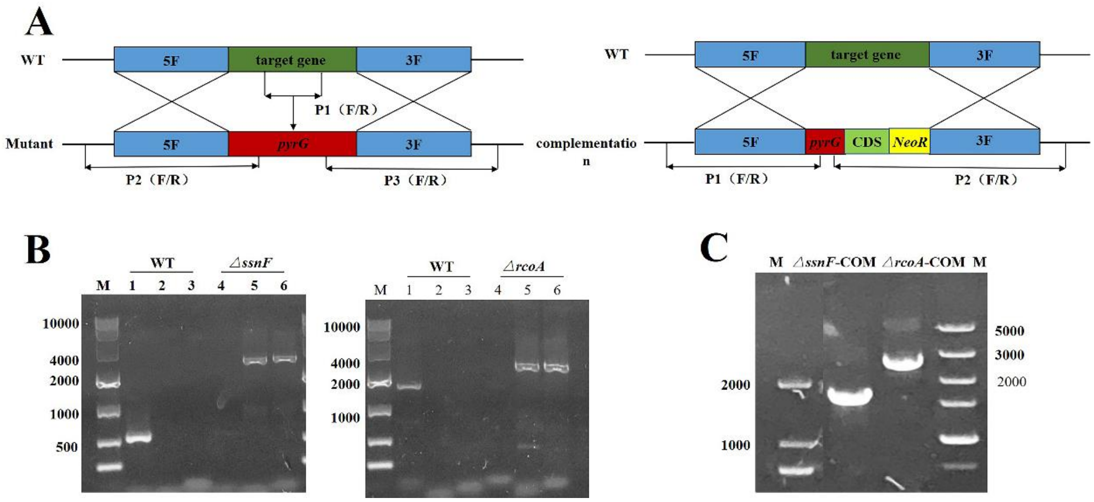

Figure S2. PCR verification of deletion and complementation strains. (A) Diagrammatic representation of the homologous recombination for the null-deletion and complementary strains constructions; (B) PCR verification for *ssnF* and *rcoA* deletion mutants; (C) PCR verification for the complementary strains.

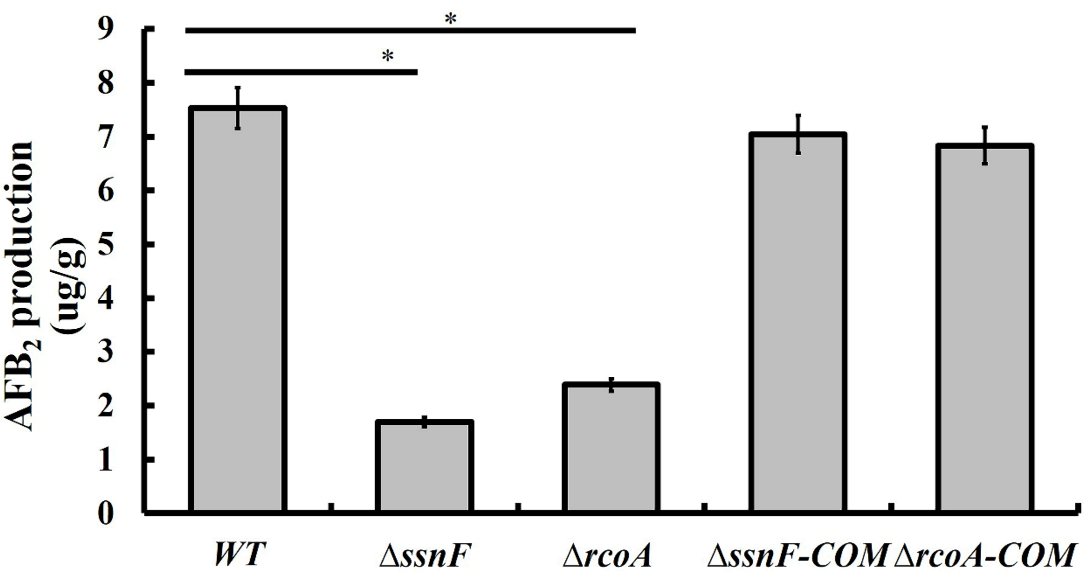

Figure S3. AFB_2 biosynthesis of different strains. Bars represent SD from three independent experiments with three replicates. * shows a significant difference at $p < 0.05$.

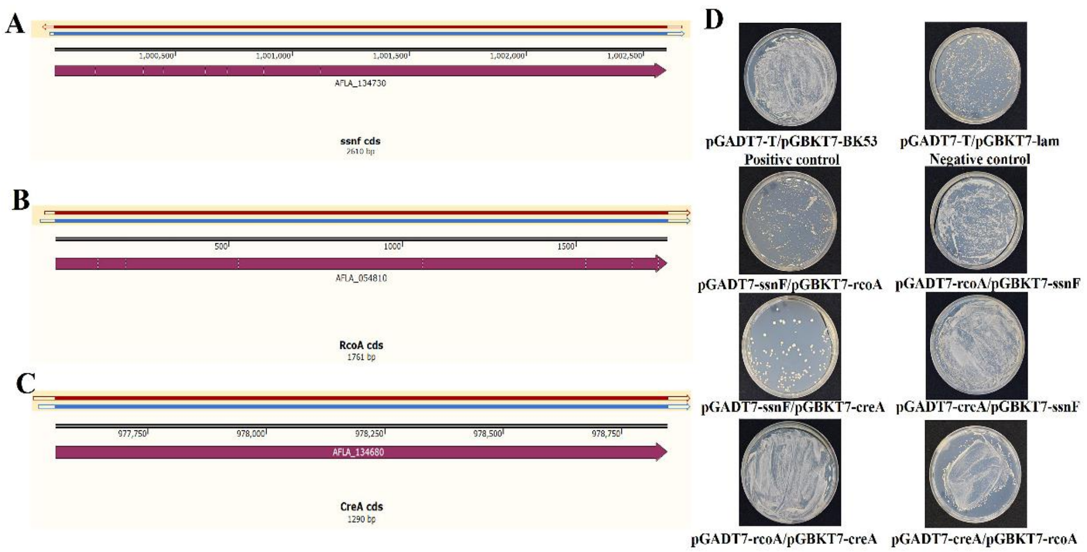

Figure S4. The acquirements of CDS fragments and the Y2H strains. (A) CDS sequence of SsnF; (B) CDS sequence of RcoA; (C) CDS sequence of CreA; (D) the Y2H strains for the interaction analyses of SsnF, RcoA and CreA. Yeast cells were co-transformed with the bait plasmid and the prey plasmids, containing the CDS regions of *ssnF*, *rcoA* and *creA* genes, respectively, and were cultivated on the nutritional deficiency plates.

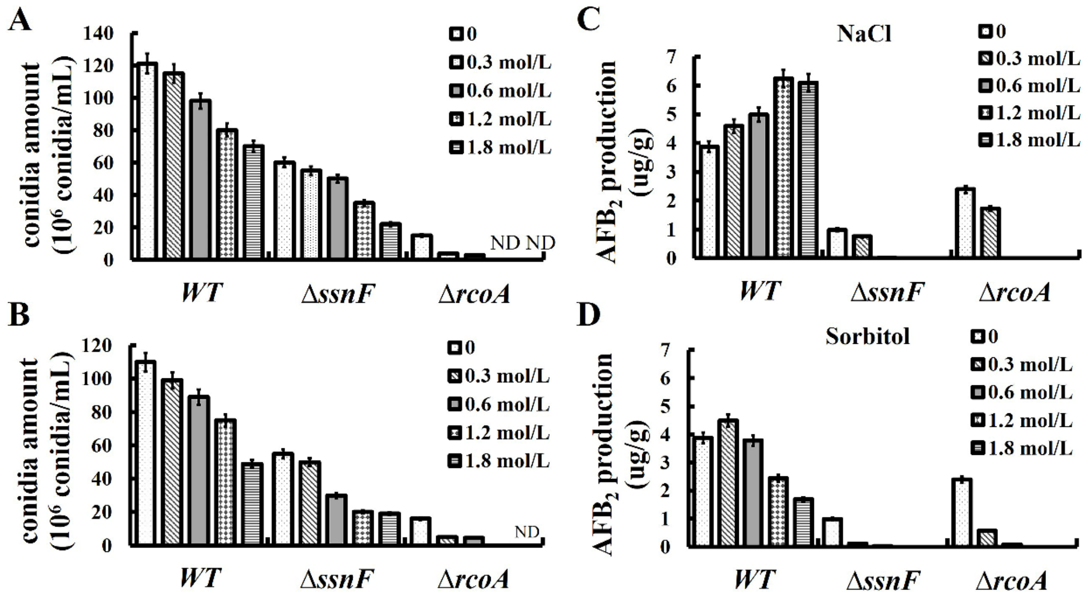

Figure S5. The productions of conidia and AFB_2 in different strains under osmotic stress. (A) conidia production of different strains with different concentrations of NaCl; (B) conidia production of different strains with different concentrations of D-sorbitol; (C) AFB_2 biosynthesis of different strains with different concentrations of NaCl; (D) AFB_2 biosynthesis of different strains with different concentrations of D-sorbitol. Bars represent SD from three independent experiments with three replicates. ND means no detected.

Table S1. PCR primers used in this study.

Name	Sequence（5′-3′）	Usage
ssnF-5F	gtcagacactgccctgtttc	Upstream homologous fragment of *ssnF* gene
ssnF-5R	tttgaagccacacctgctcggagagttattctgtgtctga	
ssnF-3F	gcctcctctcagacagaaggaagatggacgtggatgag	Downstream homologous fragment of *ssnF* gene
ssnF-3R	tctgaatcggccgcaaagtc	
ssnF-cs-F	tgcactaaggtccatgctac	Fusion segments
ssnF-cs-R	ttcggcttttcctcctgacc	
RcoA-5F	ggaccggatcgaaagaatct	Upstream homologous fragment of *rcoA* gene
RcoA-5R	tcagacacagaataactctctgttcaaactcgccagtgct	
RcoA-3F	gcctcctctcagacagaattgctcctgagaaggtttgac	Downstream homologous fragment of *rcoA* gene
RcoA-3R	aagctgactggtgtatgcac	
RcoA-cs-F	aacagatccacggagactgt	Fusion segments
RcoA-cs-R	cagggctggtcaaagatgca	
pyrG-F	gagagttattctgtgtctga	*pyrG* gene
pyrG-R	attctgtctgagaggaggc	
Checkup-R	gtcacatcagcagagacggtaac	Verification
Checkdown-F	gcttggacagcaataccagact	
SsnF-TG-F	ctgtattctgcggactaagg	Verification
SsnF-TG-R	tcaaatccattagccgcctg	
RcoA-TG-F	attctgtctgagaggaggc	Verification
RcoA-TG-R	gggaacattaagctcccaga	
NeoR-F	ctaaacaattcatccagtaaaa	Resistance gene
NeoR-R	atggctaaaatgagaatatcacc	

Table S2. qPCR primers used in this study.

Name	Sequence（5′-3′）	Usage
aflA	F: caacgccaacgctattcgag ; R: tggatgacacgttgtcccag	
aflB	F: atccactcgacatcatcgcc ; R: ttgatgtcacgtcggctgaa	
aflC	F: ctgaatcaccgaccaccgaa ; R: ccccatttggtagcccctttt	
aflD	F: gcatcggacgaggtctcatt ; R: ctgggcatcagtttccgagt	
aflG	F: tggcgtatgtggtgcagaaa ; R: ccctcagcaaacatcgaacc	
aflK	F: cgggtccttttcgacgatca ; R: ccattagcagctggggtgaa	AFs biosynthesis cluster genes
aflO	F: tgggcaaacggcaaattcag ; R: tagagttatcggcgtgtcgc	
aflP	F: tagagttatcggcgtgtcgc ; R: tgatgtgggactaggtccgt	
aflQ	F: tcgggattcgacggttcttg ; R: ctcatcttttccatgcggcg	
aflR	F: tgcagtcaatggaacacgga ; R: agcgaaaagcagcaatagcg	
aflS	F: aagtattagccctcgccagc ; R: catccagagggatacacgcc	
atfA	F: aaactgaagactcccaggcg ; R: gtaatccggggtatccgcag	
atfB	F: agcctgacctgatggctttc; R: tggggttgcaaaaggtctgt	
AP-1	F: gaagcgcagagaaagcgatg ; R: aggccttctgcaactcatcc	
srrA	F: ccttaagcccttcccaggtg ; R: ggtggctcccctaacgaatc	Diverse global regulators
mtfA	F: tactcgcaaaccccccatgac ; R: aggattcaaccggcgaagag	
msnA	F: cttgcacaccccaatcaacg ; R: gaagttgctgaccgagtcca	
veA	F: atgagacgtcggggagagaa ; R: ccgtgctggtactggttgat	
creA	F: caccggcttcttcagtggat ; R: agacgaggccatggtagagt	
creB	F: cacttgcagaggtgcgaaac ; R: gcgaagagtatttgccagcg	
creC	F: tcactcaccacctgcctcta ; R: cgccttctctcccgatcaaa	
creD	F: gcgacacttctcccgtgtat ; R: tcaactcggatgctggttcc	
snf1	F: ccctgaacccagtgcaatca ; R: tgtccggatcttgggagaga	
snf4	F: agctaagcggaaggacatcg ; R: ctctccgagaatgcctagcg	Related genes in carbon metabolism
hulA	F: cctattgtctctgcggatgct ; R: tgggttgacgaggattgtctg	
schA	F: cggactgtttgagccgatct ; R: gattgaacctccccatggca	
reg1	F: tgcgcttcctacacgacttt ; R: tcaaccggctttcccttctc	
gal83	F: ggttccagggcggagattta ; R: gttactggacccgcaagact	
rcoA	F: gagagagcccccggtaattc ; R: atctgaagctgcctcgtgtc	
ssnF	F: gtcttgcaacagcttgggtg ; R: cttggaaacctacctgcgct	
alcR	F: tatcagttctgagctcccgc ; R: ccccgggcaagtgtagtgt	
alcA	F: atacgggattttggccctcc ; R: atctgacgggttgatgcagg	
amyR	F: ctacgctttcattgccgctc ; R: cataggatgtccgtcgaggc	
amyA	F: tcgtgtcgagatggcaaaca ; R: attcgtggctgatcgtggtt	
xlnR	F: actctgctgaaaaccctccg ; R: gcatgctaaaggacgcgaag	
xlnA	F: ggtagcagagggtatgtgcc ; R: tggttgtgtgtgactgcgat	Related genes in the hydrolase
eglA	F: gatgtcgaccagggtgaagg ; R: gtccttggaggtgacatcgg	
eglC	F: gccacggttatgtgtctgga ; R: gccaatggtatctggggcat	
prnB	F: ctttgccttaggctggctct ; R: agttgatgacaaggctggca	
prnC	F: aggtcttcaagtgtctggcg ; R: agccccttgattctcaaccg	
prnD	F: cgaacaatgtgtggcagcaa ; R: ccaagcagcacacctctgta	
cbhA	F: cataccctcaacatgcaggc ; R: aacggacgacgaaggagatg	
